# Assessment of iron status among preschool children (6 to 59 months) with and without malaria in Western Province, Kenya

**DOI:** 10.11604/pamj.2015.21.62.4560

**Published:** 2015-05-28

**Authors:** Isaac Kisiangani, Charles Mbakaya, Anzelimo Makokha, Dennis Magu

**Affiliations:** 1 Institute of Tropical Medicine and Infectious Diseases, Jomo Kenyatta University of agriculture and Technology, Nairobi, Kenya; 2 Kenya Medical Research Institute, Nairobi, Kenya

**Keywords:** Serum ferritin, haemoglobin, plasmodium falciparum malaria, preschool children

## Abstract

**Introduction:**

Iron deficiency is a major public health concern. Globally, iron deficiency ranks number 9 and is responsible for about 60% of all anemia cases among preschool children. In Africa iron deficiency is 43-52% while in Kenya, children under 5 years constitute the largest burden with 69% of them being deficient. There is limited iron deficiency data in Kenya. This study determined haemoglobin levels, serum ferritin levels, nutritional status and *P.falciparum* malaria infection in preschool children.

**Methods:**

A household cross sectional study was undertaken among 125 preschoolers in Western province, drawn from 37 clusters. Systematic random sampling was used for sample selection. Data was collected using pretested structured questionnaires, entered in Microsoft package. Data analysis was done in Statistical package for social science (SPSS) version 20 using bivariate and multivariate logistic regression and differences were considered significant at P < 0.05.

**Results:**

The prevalence of iron deficiency (Serum ferritin <12mg/l), anaemia (Hb < 110g/l) and *plasmodium falciparum* malaria were 20.8%, 25% and 6.8% respectively. There was a significant association between iron deficiency and anaemia (OR = 3.43, 95% CI: 1.33-8.84, p = 0.008). A preschool child with anaemia was 3.43 times likely to be iron deficient compared to a preschool child who was not anaemic.

**Conclusion:**

Iron deficiency, anaemia and *plasmodium falciparum* malaria was prevalent among preschool children. The findings revealed a significant association between iron deficiency and anaemia. Therefore effective interventions to improve iron status will have large health benefits by greatly reducing anaemia in preschool children.

## Introduction

Globally, iron deficiency ranks number 9 and is responsible for about 60% of all anemia cases among preschool children. The global burden of iron deficiency has been estimated from anaemia prevalence surveys and is undoubtedly large. Iron deficiency is a significant public health problem in Kenya among preschool children because their bodies need iron to grow and develop. Its control is a global priority in public health [1]. Anemia due to iron deficiency affects up to 60% of all children globally [2, 3]. The WHO estimates 27% of preschool children suffer from anaemia due to iron deficiency [4].

In the developing world, 42% of children less than five years of age are highly affected [5]. The largest burden of anaemia is in children under 3 years of age, pregnant and lactating women [6]. Iron deficiency is the main cause of anaemia accounting for about 50% of all anaemia cases [7, 8]. Iron deficiency anaemia results from a variety of causes including inadequate iron intake, high physiologic demands in early childhood and iron losses from parasitic infections, especially malaria, are important factors contributing to the high prevalence of anaemia in many populations. Iron deficiency alone, with or without anemia, lead to defects in neurodevelopment and delays in acquisition of motor and mental milestones these effects persist into middle childhood and adolescence [9].

The aim of this study was to determine iron status, nutritional status and *P.falciparum* malaria infection rate and factors affecting iron deficiency among preschool children in Western Province. The objective was to describe iron deficiency by anaemia, age, sex, residence, wealth index, anthropometric indicators and malaria infection.

## Methods

**Study site:** The study was carried out in Western Province of Kenya outside Nairobi, West of the Eastern Rift Valley and is inhabited mainly by the Luhya people. It harbours 3,358,776 inhabitants within an area of 8,361 km^2^. The main economic activity is farming with maize as the staple food. This area is a stable malaria endemic area.

**Study population:** The study population consisted of preschool children aged between 6-59 months. The inclusion of the study subjects was based on consenting parents/ guardians of children aged 6-59 months and without physical disability that would affect height measurement but those on iron supplementation, blood transfusion and had physical disability were excluded from the study.

**Study design and sampling procedure:** The study was a cross-sectional that used a two-stage stratified cluster sample. The households were clustered using National Sample Survey & Evaluation Programme (NASSEP IV). The province was stratified into rural and urban EAs (enumeration areas). The first stage involved selection of Primary Sampling Units (PSUs) which were the EAs using probability proportional to measure of size (PPMOS) method. The second stage involved the selection of households and EAs were selected with a basis of one measure of size (MOS) defined as the ultimate cluster with an average of 100 households and constituted of one (or more) EAs. The household and structure were listed through a quick count, amalgamation /segmentation of EAs to form clusters, physical numbering of the structure of the dwelling unit.

The sample was selected using a stratified two-stage cluster design consisting of 37 clusters, 18 in the urban and 19 in the rural areas. For each cluster a total of 10 households were selected using systematic simple random sampling.

### Data collection

***Clinical history, demographic and socioeconomic data:*** A structured questionnaire was used to record data. It captured demographic, socioeconomic, anthropometric measurements, health history, knowledge and behaviours on children aged 6-59 months.

***Sample collection and transportation:*** Venous blood sample was drawn into heparin and EDTA tubes for determination of Haemoglobin, malaria and serum ferritin concentration and stored in a cool box containing gel packs, then transported to cluster lab and processed. Finger stick or heels stick blood collection procedures was only used in cases of collapsed veins or very small veins. The blood collected in the heparin tube was centrifuged at 3000 rpm for 10 minutes to obtain serum at a central field lab site. The serum was frozen at -200c for transportation to the central laboratory and analyzed within 1 month of blood collection.

**Iron status indexes:** Haemoglobin concentration Haemoglobin was determined from venous blood sample in EDTA tube using "Hemocue globinometer" (Hemocue HB-301). Anemia was defined as Hb below 11.0g/dl. *Serum Ferritin*The serum ferritin concentration was determined with Elegance Amplified Enzyme Linked Immunosorbent Assay (ELISA) system. Iron deficiency (ID) was defined as serum ferritin below 12mg/dl [10] but a limitation during infection where serum ferritin is elevated. A secondary analysis was performed using Thurnham's et al, proposed correction factors.

***Malaria screening:*** Malaria rapid diagnostic kits (RDKs) were used at the household for malaria test using blood collected in EDTA tubes. The RDKs used were *P. falciparum* only (HRP2) to capture *P. falciparum* malaria. Thick blood smears was prepared on glass slides within 2 hours of blood collection. The slides were fixed and stained with Giemsa stain and allowed to dry and observed under a microscope using oil immersion objectives (x100). The presence or absence of malaria was reported as any parasiteamia detected in blood smear

***Anthropometric:*** Height and weight were measured among children who were in light clothing to determine their nutritional status. The weight measurement was taken using a Seca scale (Hanson mode) to the nearest 0.1 kg and height/ length portable wooden constructed scale calibrated for height measurement to the nearest 0.1 cm. Height for age (stunting), weight for age (underweight) and weight for height were calculated using = 2D NCHS (National Center for Health Statistics) reference data. The height for age Z-score (HAZ) of <-2 was classified as stunted and Z-score cut off point of <-2SD was used to classify low weight for age, low height for age and low weight for height.

### Statistical analysis

The data was coded and double entered into a computer database using Ms-Access and Ms-Excel and analysis was performed using SPSS. In order to maintain the assumption of an equal probability sample, weighting was used to adjust for unequal cluster size due to variation in the number of absentees or refusals between clusters. The main predictor, iron deficiency, was defined as serum ferritin <12 µg/L. Possible confounders considered for inclusion were: sex, age, area of residence, socio economic status, malaria, nutritional status and anemia. Bivariate analysis was performed using Chi-square to determine the association between dependent variable and independent variables. A multivariate logistic regression using backward method was used to explore determinants of iron deficiency and P-values <0.05 were considered statistically significant.

### Ethical clearance

Ethical clearance was sought from scientific steering committee (SSC) and ethical review committee (ERC) of Kenya Medical Research Institute (KEMRI) for approval. Prior consent was sought from parents/guardians of the preschoolers who participated in the study. During the interview privacy and confidentiality was observed.

### Limitation

The surveys produced the province estimates, and for a subset of indicators, cluster estimates that could not be generalized. This consideration was based on the prohibitive costs of taking a representative sample within the province. In specific population certain indicators would not be assessed, such as hook worm infestation and other infections in preschoolers due to feasibility and cost. The cross-sectional nature of this survey did not allow for measurement of iron level indicators over time, and might have been influenced by seasonality, illness and other external factors present at the time of survey collection. Socio-cultural beliefs about taking of blood samples still prevailed in some areas of the study.

## Results

### Selected demographic characteristics of the participants

A total of 125 preschoolers aged 6-59 months were enrolled in the study and consisted of males 72 (57.6%) and 53 (42.4%) females with a mean age of 35+ (10 SD) ranging between 6-59 months. A high proportion (29.6%) was aged between 24-35 months. The majority (65.6%) of the participants resided in rural areas and a substantive proportion (34.4%) in urban areas as shown in Table 1.

**Table 1:** Iron deficiency in relation to selected demographic characteristics

Variables	Sample size		Deficient (n = 26)		Normal (n = 99)		OR	95% CI		p value
	n = 125	%	n	%	n	%		Lower	Upper	
**Age in months**										
6-11 months	5	4	1	20.00%	4	80.00%	1.2	0.11	13.15	0.881
12-23 months	18	14.4	7	38.90%	11	61.10%	3.05	0.79	11.8	0.105
24-35 months	37	29.6	7	18.90%	30	81.10%	1.12	0.32	3.98	0.861
36-47 months	36	28.8	6	16.70%	30	83.30%	0.96	0.26	3.53	0.951
48-59 months	29	23.2	5	17.20%	24	82.80%	1			
**Sex**										
Male	72	57.6	19	26.40%	53	73.60%	2.36	0.91	6.11	0.073
Female	53	42.4	7	13.20%	46	86.80%	1			
**Residence**										
Rural	82	65.6	14	17.10%	68	82.90%	0.53	0.22	1.28	0.156
Urban	43	34.4	12	27.90%	31	72.10%	1			

### Household economic characteristics of mothers/guardians

**Figure 1:**
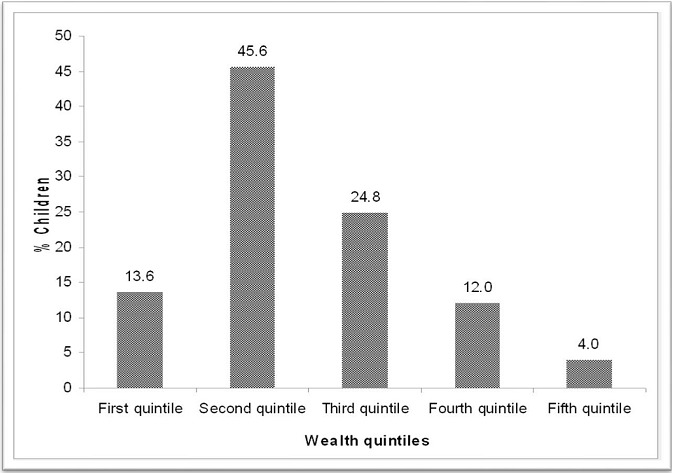
Household wealth index

The wealth was defined by the type of house, roofing material and number of sleeping rooms as revealed in Table 2. The study findings indicated that the main material of the (inside) walls of the house was mud (69.6%) and main house roofing materials was corrugated iron (88.8%). The commonly used source of energy for cooking was wood as reported by 72.8% of the participants. The other sources of energy for cooking were LPG/natural gas (4%), charcoal (22.4%) and others (0.8%). The economic status for each household was determined by means of a wealth index, which was a generic of all the social economic characteristics. Going by the wealth index scale, the bulk of the population (45.6%) were in the second quintile. The minority of the population were in the fifth quintile (4%) as shown in Figure 1.

**Table 2:** Household economic characteristics

Variables	n = 125	%
**Main Material of the floor of the house**		
Earth/ sand	36	28.8
Dung	54	43.2
Cement	35	28
**Main Material of the roof of the house**		
Grass / thatch / makuti/ Dung / mud	12	9.6
Corrugated iron (mabati)	111	88.8
Asbestos sheet	2	1.6
**Main material of the (inside) walls of the house**		
Dirt/Mud/Dung	87	69.6
Bamboo with mud/ Stone with mud	4	3.2
Cement	30	24
Bricks	3	2.4
Cement blocks	1	0.8
**Household ownership**		
Clock/watch	36	28.8
Electricity	13	10.4
Radio	97	77.6
Television	30	24
Mobile Telephone	91	72.8
Fixed Telephone	2	1.6
Refrigerator	7	5.6
Solar Panel	4	3.2
**Type of fuel used for cooking**		
LPG/natural gas.	5	4
Charcoal	28	22.4
Wood	91	72.8
Other	1	0.8
**Where cooking is usually done**		
In the house	41	32.8
In a separate building.	78	62.4
Outdoors.	6	4.8
**Number of rooms used for sleeping**		
One	57	45.6
Two	47	37.6
Three	16	12.8
Four	5	4

### Iron deficiency, anaemia, nutritional status and malaria status

**Figure 2:**
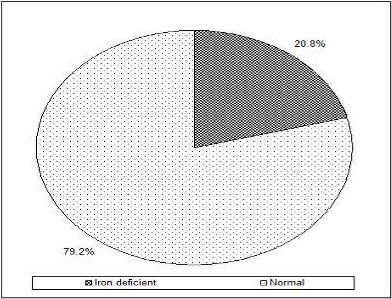
Iron deficiency status

The prevalence of iron deficiency in children with serum ferritin concentrations <12 mg/L was 20.8% (Figure 2). Stunting (Height for age = 2D, 28.9%) and wasting (WHZ = 2SD, 1.7%) were prevalent. The Hb cut off point was 11.0g/dl. Thirty children (25%) were anaemic and malaria prevalence was 6.7% as shown in Table 3.

**Table 3:** Anemia, nutrition status and malaria status

Variables	n = 125	%
**Anemia status**		
Anemic	30	25
Normal	90	75
Non-response	5	
**Malaria status**		
Positive	7	6.7
Negative	98	93.3
Non-response	20	
**Nutrition Status**		
Underweight	8	6.6
Stunting	36	28.9
Wasting	2	1.7

### Bivariate analysis

The selected demographic characteristics were age in months, sex and residence that were not significantly associated with iron deficiency. Using children aged 48-59 months as the reference, males in all the age groups were at a higher risk of iron deficiency than females (26.4%, OR = 2.36, 95% CI: 0.91-6.11, p = 0.073). The risk of iron deficiency decreased with age increase among the subjects, progressively lowering with each age group (Table 1). Using fourth / fifth quintiles as the reference for the relationship between iron deficiency and household economic characteristics, none of the wealth index was significantly associated with iron deficiency (Table 4). Interestingly none of the anthropometric measures were associated with iron deficiency. A low proportion of the preschooler (37.5%) who were underweight were iron deficient (OR = 2.63, 95% CI: 0.58-11.87, p = 0.194). Among the subjects who had stunted growth, a small proportion (20%) had iron deficiency as compared to those stunted with normal iron status (OR = 1.01, 95% CI: 0.38-2.71, p = 0.977). The iron deficient cases in preschoolers who were wasted could not be determined (Table 5). There was a significant association between iron deficiency and anaemia as subjects who were iron deficient, 36.7% of them were anaemic (OR = 3.43, 95% CI: 1.33-8.84, p = 0.008) as shown in Table 5. There was no association between iron deficiency and malaria as subjects who were malaria positive, 14.3% were iron deficient compared to who were malaria positive with normal iron status (83.7%), (OR = 0.54, 95% CI: 0.06-4.75, p = 0.576).

**Table 4:** Iron deficiency in relation to household economic characteristics

Variables	Deficient (n = 26)		Normal (n = 99)		OR	95% CI		p value
	n	%	n	%		Lower	Upper	
**Wealth index**								
First quintile	7	41.20%	10	58.80%	2.1	0.52	8.51	0.299
Second quintile	7	12.30%	50	87.70%	0.42	0.12	1.52	0.186
Third quintile	7	22.60%	24	77.40%	0.88	0.23	3.26	0.842
Fourth/ Fifth quintile	5	25.00%	15	75.00%	1			

**Table 5:** Iron deficiency in relation to anaemia, nutrition status and malaria prevalence among the children

Variables	Deficient (n = 26)		Normal (n = 99)		OR	95% CI		p value
	n	%	N	%		Lower	Upper	
**Anaemia status**								
Anaemic	11	36.70%	19	63.30%	3.43	1.33	8.84	**0.008**
Normal	13	14.40%	77	85.60%	1			
Not tested	2		3					
**Malaria status**								
Positive	1	14.30%	6	85.70%	0.54	0.06	4.75	0.576
Negative	23	23.50%	75	76.50%	1			
Not tested	2		18					
**Underweight**								
Underweight	3	37.50%	5	62.50%	2.63	0.58	11.87	0.194
Not underweight	21	18.60%	92	81.40%	1			
**Stunting**								
Stunted	7	20.00%	28	80.00%	1.01	0.38	2.71	0.977
Not stunted	17	19.80%	69	80.20%	1			
**Wasting**								
Wasted	0	0.00%	2	100.00%	UD	UD	UD	1
Not wasted	24	20.20%	95	79.80%	1			

### Multivariate analysis

Multivariate analysis was performed to identify independent variables. The four variables included (1) Sex, (2) Consumed juice or juice drinks in the last 24hrs, (3) Consumed sour milk, cheese, yoghurt or other food made from milk in the last 24hrs and (4) anaemia status. Upon fitting the factors using Binary logistic regression and specifying ‘backward conditional’ method with removal at P < 0.05, One factor was retained in the final model as shown in Table 6. There was a significant association between iron deficiency and anaemia (OR = 3.43, 95% CI: 1.33-8.84, p = 0.008). A preschooler with anaemia was 3.43 times likely to be iron deficient compared to the non anaemic subjects

**Table 6:** Factor(s) associated with iron deficiency in children

Variables	OR	95% CI		p value
		Lower	Upper	
**Full model**				
**Sex**				
Male	1.60	0.58	4.44	0.368
Female	1.00			
**Consumed Juice or juice drinks in the last 24 hours**				
No	0.28	0.07	1.17	0.082
Yes	1.00			
**Consumed Sour milk, cheese, yoghurt or other food made from milk in the last 24 hours**				
No	0.17	0.02	1.31	0.089
Yes	1.00			
**Anaemia status**				
Anaemic	2.79	1.02	7.65	**0.046**
Normal	1.00			
**Reduced model**				
**Anaemia status**				
Anaemic	3.43	1.33	8.84	**0.008**
Normal	1.00			

## Discussion

The study findings indicated that iron deficiency was prevalent at 20.8% when low serum ferritin concentration (<12mgl^-1^) was taken into account (Figure 2). The findings were consistent to the findings in a cross-sectional study done among adolescent school girls in western Kenya where iron deficiency was 19.8% [11]. According to Zimmerman, the low prevalence of ID based on ferritin in this study may have been found in children with depleted iron stores who were yet to progress to iron deficient erythropoeisis [12]. This study measured many factors thought to be associated with iron deficiency including anemia among preschoolers in a developing country setting where malaria is endemic. Association of non-modifiable characteristics including sex, residence and age (6-59 months) with iron deficiency was also determined. Iron deficiency in malaria endemic regions has multiple causes of which p.falciparum being one of the causes [13]. In this study the prevalence of malaria parasitaemia reported was 6.7% and this was lower than 60% reported by Fuseni in Ghana [14]. The findings showed no association between malaria and iron deficiency. This finding differs with previous findings where malaria is implicated as the major cause of iron deficiency in young children [15]. This might be attributed to low prevalence of malaria in the study population probably as a result of the existence of an active control program [16]. The declined malaria cases might have resulted from intensified interventions that included scaled up use of insecticide-treated nets including long lasting insecticides nets (ITNs), artemisinin-combination therapy (ACT) and indoor residual spraying (IRS) [17, 18].

The 25% prevalence of anemia in this study is consistent with 28% reported by Ndyomugyenyi in Masindi Uganda [19]. This was in contrast with the Kenya National Micronutrient report of 1999 where the prevalence of anemia was 69% in children 6- 59 months. It is possible that one or more other nutrients deficiencies linking stunting to erythtopoiesis. In respect to anemia, the present study found a significant relationship between iron deficiency and anemia (p = 0.008). The results showed that children who were anemic were more likely to have abnormal iron status values. A preschool child with anemia was 3.43 times likely to be iron deficient. This finding concurs with other research findings where traditionally prevalence of anemia has been used to estimate the prevalence of iron deficiency and iron deficiency anemia [20, 21]. It is also consistent with research findings in Nyando District, Kenya among preschoolers where iron deficiency was associated with anemia [22]. The findings from this study are similar to those of Kadivar in Fars province [23] and Karimi [24] in Yazd province in central Iran. In both studies there was no significant association between iron deficiency and sex. Similar studies in other parts of the world reached the same conclusion [25]. Although the results of this study showed no significant relationship (p > 0.05) between sex and iron deficiency, it was found that males were at a higher risk of iron deficiency (26.4%, OR = 2.36, 95% CI: 0.91-6.11) than female children. This is consistent with Domellof who concluded that infant boys were at a higher risk of iron deficiency. They suggest the reason for this is that boys may be born with smaller iron resources because of their higher birth weight or may have more infections than girls [26].

The current study also highlighted the impact of socioeconomic status on iron status. There was no association between iron deficiency and economic status. This differs with a study in Thailand that observed a higher risk of acquiring IDA in low income cases [27]. Likewise, a study conducted in West Malaysia also highlighted the impact of low socioeconomic status on iron status among rural children [28].

## Conclusion

Iron deficiency, anemia and *plasmodium falciparum* malaria was prevalent among preschoolers (aged 6-59 months). The prevalence of ID is same in both sexes. The findings revealed a significant association between iron deficiency and anemia. Therefore effective interventions to improve iron status will have large health benefits by greatly reducing anemia in preschool children.
